# Network pharmacology and experimental validation methods to reveal the active compounds and hub targets of *Curculigo orchioides* Gaertn in rheumatoid arthritis

**DOI:** 10.1186/s13018-023-04352-w

**Published:** 2023-11-13

**Authors:** Xia Liu, Mingchun Huang, Lijuan Wang, Jie Li, Weihui Wu, Qin Wang

**Affiliations:** Department of Pharmacy, Chongqing Traditional Chinese Medicine Hospital, No. 6 Panxi Qizhi Road, Chongqing, 400021 China

**Keywords:** Rheumatoid arthritis, *Curculigo orchioides* Gaertn, Network pharmacology, Molecular docking

## Abstract

**Background:**

Rheumatoid arthritis (RA) is an autoimmune disease that can lead to joint destruction and deformity. *Curculigo orchioides* Gaertn (CO) was previously revealed to play a significant role in RA treatment. However, the main active ingredients and molecular mechanisms of CO in regulating RA are still unclear.

**Methods:**

The active ingredients of CO were obtained from the Traditional Chinese Medicine Systems Pharmacology database and published literature. The targets corresponding to these compounds and the targets linked to RA were collected from public databases. The “ingredient-target” and “protein–protein interaction” networks were constructed to screen the main active ingredients and hub targets of CO in the treatment of RA. Gene Ontology and Kyoto Encyclopedia of Genes and Genomes enrichment assays were used to elucidate the potential pharmacological mechanism of CO in RA. Molecular docking was performed to detect the binding between the main active ingredients and hub targets. Collagen-induced arthritis rats were used to validate the hub targets of CO against RA.

**Results:**

Network pharmacological topology analysis showed that caffeine, 2,4-dichloro-5-methoxy-3-methylphenol, curculigoside, orcinol glucoside, and orcin were the main active ingredients of CO, and matrix metalloproteinase 9 (MMP9), transcription factor AP-1 (JUN), prostaglandin-endoperoxide synthase 2 (PTGS2), brain-derived neurotrophic factor, and receptor-type tyrosine-protein phosphatase C were the hub targets of CO for RA treatment. Molecular docking revealed that curculigoside and orcinol glucoside had effective binding potential with MMP9, JUN, and PTGS2, respectively. In vivo experiments demonstrated that CO alleviated RA symptoms and inhibited the expression of MMP9, JUN, and PTGS2 proteins.

**Conclusions:**

Our study demonstrates the main active ingredients and potential targets of CO against RA, laying an experimental foundation for the development and application of CO as an anti-RA drug.

## Introduction

Rheumatoid arthritis (RA) is a chronic autoimmune disease featured by the inflammation of tendon, finally triggering cartilage destruction, bone erosion, and even joint damage, deformity, and disability [1]. The global prevalence of RA is estimated to be about 0.27% [2]. Age, gender, genetics, and environmental exposure are the main risk factors for RA [3]. Nonsteroidal anti-inflammatory drugs (NSAIDs), disease-modifying antirheumatic drugs (DMARDs), glucocorticoids, and biologic agents are the main medications used for RA treatment [4]. Although these drugs can improve the disease course and relieve the clinical manifestations, they also give rise to some unfavorable side effects including liver and kidney injury and cardiovascular risk [5]. Thus, it is necessary to explore more safe and effective drugs to treat RA.

Traditional Chinese medicine (TCM) has gained rising attention in RA treatment due to its excellent effectiveness and low toxicity [6, 7]. According to the TCM theory, RA belongs to the category of “Bi Zheng” (bi-syndrome), and the core pathogenesis of bi-syndrome is kidney deficiency [8]. *Curculigo orchioides* Gaertn (CO) is a perennial herb traditionally used to nourish the kidney, strengthen muscles and bones, and dispel cold and dampness [9]. CO and its preparations are extensively applied in clinical practice and have various pharmacological properties, such as anti-osteoporosis [10], anti-oxidant [11], anti-diabetic [12], and anti-inflammatory activities [13]. Our previous research has shown that wine-processed CO (pCO), one of the processed products of CO, has a stronger effect in resisting RA than CO [14]. It is likely attributed to the quantitative differences of the main active ingredients, which are detected by the ultra-high-performance liquid chromatography Q exactive mass, from pCO and CO. However, the current research reports and clinical applications still focus on CO. Therefore, this study aims to explore the main active ingredients of CO against RA and its molecular mechanism from the perspective of network pharmacology, which will provide a basis for the clinical applications of CO and further elucidation of the mechanism of pCO in the later stage.

**Fig. 1:**
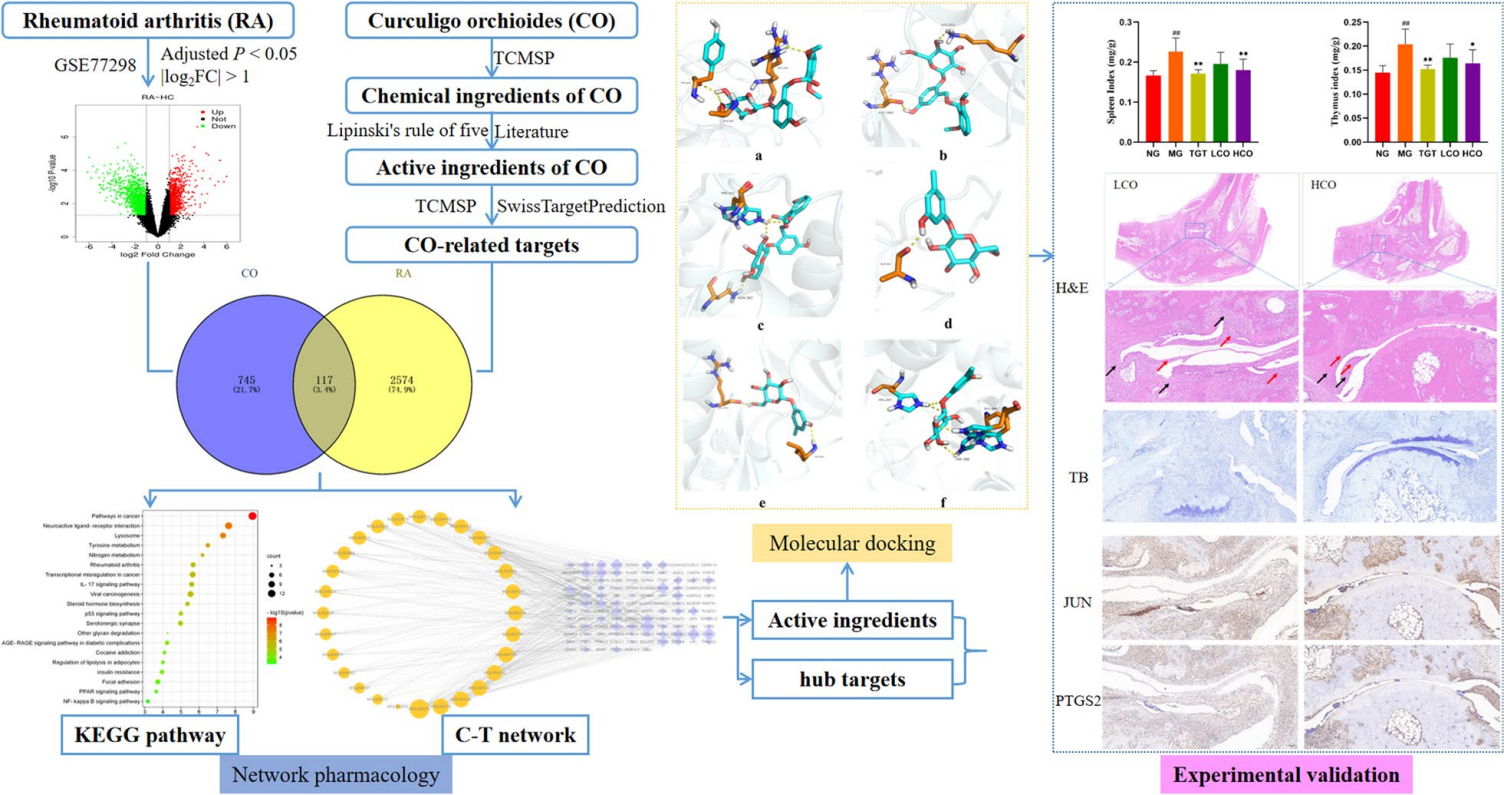
Flowchart of CO in the treatment of RA

Network pharmacology is a system-level approach integrating pharmacology, network biology, and bioinformatics to elucidate the links among drugs, targets, and diseases [15]. Given the multi-component, multi-target, and multi-pathway nature of TCM, network pharmacology is becoming ubiquitous in TCM research to screen active substances, predict potential targets, and reveal specific mechanisms [16]. Therefore, in this study, we attempted to identify anti-RA active ingredients and potential targets for CO by network pharmacology, molecular docking, and subsequent experimental validation (Fig. 1).

## Materials and methods

## Network pharmacology analysis

### Screening active ingredients from CO

The chemical compounds of CO were acquired from the TCMSP database (https://www.tcmspw.com/). We applied Lipinski’s rule of five as the standard to screen the active ingredients of CO [17]. Compounds in CO that have been reported to have anti-RA activities in the published literature were also included.

### Collecting potential targets of active ingredients

The obtained active ingredients were imported into the PubChem database (https://pubchem.ncbi.nlm.nih.gov/) to query the canonical SMILES string, and active ingredients without a canonical SMILES string were deleted. SwissTargetPrediction (http://new.swisstargetprediction.ch) and Traditional Chinese Medicine Systems Pharmacology (TCMSP) databases were utilized to retrieve the potential targets of active ingredients. After removing repeating targets from the two databases, we obtained the potential targets of CO.

### Differentially expressed genes (DEGs) analysis

GSE77298 gene expression profiles were downloaded from the GEO database (https://www.ncbi.nlm.nih.gov/geo/) with the keyword “rheumatoid arthritis” and species “Homo sapiens.” This dataset contains samples from 7 healthy controls and 16 RA patients. The limma package of R was applied to screen DEGs between RA patients and healthy controls. Adjusted *P* < 0.05 and |log_2_Fold Change (FC)|> 1 were set as the screening thresholds. The DEGs were presented as the volcano plot, and the heat map of the expression data was generated.

### Network establishment

The harvested DEGs and targets of active ingredients were overlapped to generate targets related to both RA and CO. Then, the overlapping targets were submitted into the STRING database (https://string-db.org/) to build a protein–protein interaction (PPI) network with species restricted to “Homo sapiens” and a confidence score ≥ 0.4. Finally, the PPI network was visualized and analyzed by Cytoscape 3.7.1 software (https://www.cytoscape.org/).

To elucidate the relationship between the ingredients and their corresponding targets, we established a “active ingredient-target” using Cytoscape software. Finally, the hub targets and the top five active ingredients were obtained according to the degree values.

### GO and KEGG enrichment analyses

The functions and pathways of potential targets were systematically investigated by Gene Ontology (GO) annotation and Kyoto Encyclopedia of Genes and Genomes (KEGG) pathway enrichment analyses using the Metascape database (https://metascape.org/gp/index.html#/main/step1) with the species set as “Homo sapiens.” The enrichment items with *P* < 0.05 were regarded as statistically significant.

## Molecular docking

Molecular docking was performed to assess the interaction between the top five active ingredients and the hub targets. Briefly, the 3D molecule structures of ingredients were acquired from the PubChem database. The protein crystal structures of hub targets collected from the RCSB PDB database (http://www.rcsb.org/) were imported to the PyMOL software to delete the water molecules and separate the ligand. AutoDock Tools 1.5.6 was utilized to convert the small molecules and target proteins to pdbqt format. Autodock Vina software was used for molecular docking [18]. Finally, results were visualized and analyzed by the PyMOL tool.

## Experimental validation

### Chemicals and reagents

CO was obtained from Sichuan Neautus Traditional Chinese Medicine Co. Ltd (Chengdu, China) and authenticated by Prof. Ling Chen (Chongqing Traditional Chinese Medicine Hospital). Bovine type II collagen, complete Freund’s adjuvant (CFA), and incomplete Freund’s adjuvant (IFA) were acquired from Chondex Inc. (Redmond, USA).

### Preparation of CO samples

The precisely weighed dry CO powder was added to the conical flask, followed by heat reflux extraction with 70% ethanol for 2 h [19]. The extracts of CO were filtrated and concentrated to 1 g crude drug/mL. To control the quality for CO, the contents of curculigoside and orcinol glucoside were measured using high-performance liquid chromatography [20].

### Animals

Male Wistar rats (weighing 160–180 g) were supplied by Beijing Vital River Laboratory Animal Technology Co., Ltd. (Beijing, China). The Ethics Committee of Chongqing Traditional Chinese Medicine Hospital approved the experimental protocols (Permit NO. 2020KY-LX). The rats were bred in an air-conditioned room at a constant temperature (23 ± 1 °C) and humidity (60 ± 5%) and were given free access to food and water.

### Collagen-induced arthritis (CIA) induction and CO treatment

Bovine type II collagen was dissolved in an equal amount of CFA or IFA in an ice bath. Rats were randomly assigned into five groups (*n* = 8): normal group (NG), model group (MG), tripterygium glycosides (TGT, 9 mg/kg), low-dose CO group (LCO, 3 g crude drug/kg), and high-dose CO group (HCO, 7 g crude drug/kg). Rats in the MG, TGT, LCO, and HCO groups were immunized subcutaneously at the base of the tail with 0.2 mL CII-CFA emulsifier. Seven days later, a booster immune was administered near the primary injection site with 0.1 mL CII-IFA emulsion. The NG rats were injected with an equal volume of normal saline. Drug intervention was performed subcutaneously from day 14 until day 42. The 0.5% sodium carboxymethylcellulose (CMC-Na) solution was taken as a vehicle for administration. Rats in the NG and MG groups were subcutaneously administered with the same volume of saline.

### Measurement of viscera index in CIA rats

On day 42, all rats were sacrificed by cervical dislocation. The spleen and thymus were removed, washed with saline, and weighed. The viscera index was calculated according to the formula: Viscera index (mg/g) = viscera weight (mg)/body weight (g).

### Histopathologic evaluation

The ankle joints were fixed with 4% paraformaldehyde solution, decalcified with 10% EDTA, embedded in paraffin, and cut into 5-μm-thick slices. Sections were stained with hematoxylin and eosin (H&E) and toluidine blue (TB). A light microscope was used to investigate the inflammatory cell infiltration of synovium, pannus formation, and progressive destruction of articular cartilage.

### Immunohistochemical staining

Sections were immersed in 3% hydrogen peroxidase to block endogenous peroxidase, and then incubated overnight at 4 °C with primary antibodies against MMP9 (Servicebio, Wuhan, China, 1:500), JUN (Servicebio, Wuhan, China, 1:500), and PTGS2 (Servicebio, Wuhan, China, 1:500). Specimens were incubated with corresponding secondary antibodies, stained with 3,3-diaminobenzidine (DAB) substrate, and further counterstained with hematoxylin.

## Statistical analysis

GraphPad Prism 8.0 software was applied to analyze all data. The results were presented as mean ± standard deviation (SD). One-way analysis of variance (ANOVA) was used for group comparison and *P* < 0.05 denoted a statistically significant difference.

## Results

### Screening of active ingredients and putative targets from CO

A total of 35 active ingredients conforming to Lipinski's rule were collected through the TCMSP database, and seven active ingredients without canonical SMILES strings were deleted. Curculigoside is reported to have therapeutic effects against RA [21–23], so a total of 29 active ingredients (Table 1) were screened for subsequent studies. We obtained 128 and 822 targets of active ingredients from TCMSP and SwissTargetPredicition databases, respectively. After removing the duplicates from the two databases, we acquired 862 targets of CO.

**Table 1:** Potential active ingredients of CO

No	Mol ID	Molecule name	MW	AlogP	Hdon	Hacc
1	MOL000127	Neral	152.26	3.19	0	1
2	MOL002031	Toluene	92.15	2.32	0	0
3	MOL002202	Tetramethylpyrazine	136.22	0.66	0	2
4	MOL002689	3,4,5-Trimethoxytoluene	182.24	2.27	0	3
5	MOL000396	(+)-Syringaresinol	418.48	2.1	2	8
6	MOL003973	Caffeine	194.22	− 0.1	0	5
7	MOL004113	(E)-6-Methyl-3,5-heptadien-2-one	152.26	2.89	0	1
8	MOL004114	3,2′,4′,6′-Tetrahydroxy-4,3′-dimethoxychalcone	332.33	2.6	4	7
9	MOL004119	5-METHYLFURFURAL	110.12	1.13	0	2
10	MOL004121	2,4,6-Trichloro-3-methoxy-5-methylphenol	241.5	4.03	1	2
11	MOL004127	Curcumadiol	238.41	2.56	2	2
12	MOL004130	TETRAMETHYLSUCCINAMIDE	172.26	-0.79	0	4
13	MOL004132	1-Bromo-2-methoxynaphthalene	237.1	3.47	0	1
14	MOL004133	Orcinol glucoside	286.31	− 0.12	5	7
15	MOL004134	Orcin	124.15	1.78	2	2
16	MOL004136	Lycorine	287.34	0.71	2	5
17	MOL004137	Yuccagenin	430.69	3.67	2	4
18	MOL004140	2,4-dichloro-5-methoxy-3-methylphenol	207.06	3.36	1	2
19	MOL004162	Curculigenin A	474.8	4.18	3	4
20	MOL004163	Curculigenin B	476.82	4.36	4	4
21	MOL004167	2,6-Dimethoxybenzoic acid	182.19	1.4	1	4
22	MOL004169	2-PROPYL-1-HEPTANOL	158.32	3.57	1	1
23	MOL004170	DBQ	220.34	3.13	0	2
24	MOL000714	Hyacinthin	120.16	1.52	0	1
25	MOL000718	3-Methoxyanisole	138.18	1.8	0	2
26	MOL003578	Cycloartenol	426.8	7.55	1	1
27	MOL000358	Beta-sitosterol	414.79	8.08	1	1
28	MOL000449	Stigmasterol	412.77	7.64	1	1
29	MOL004126	Curculigoside	466.48	0.80	5	11

### Identification of RA-related DEGs

**Fig. 2:**
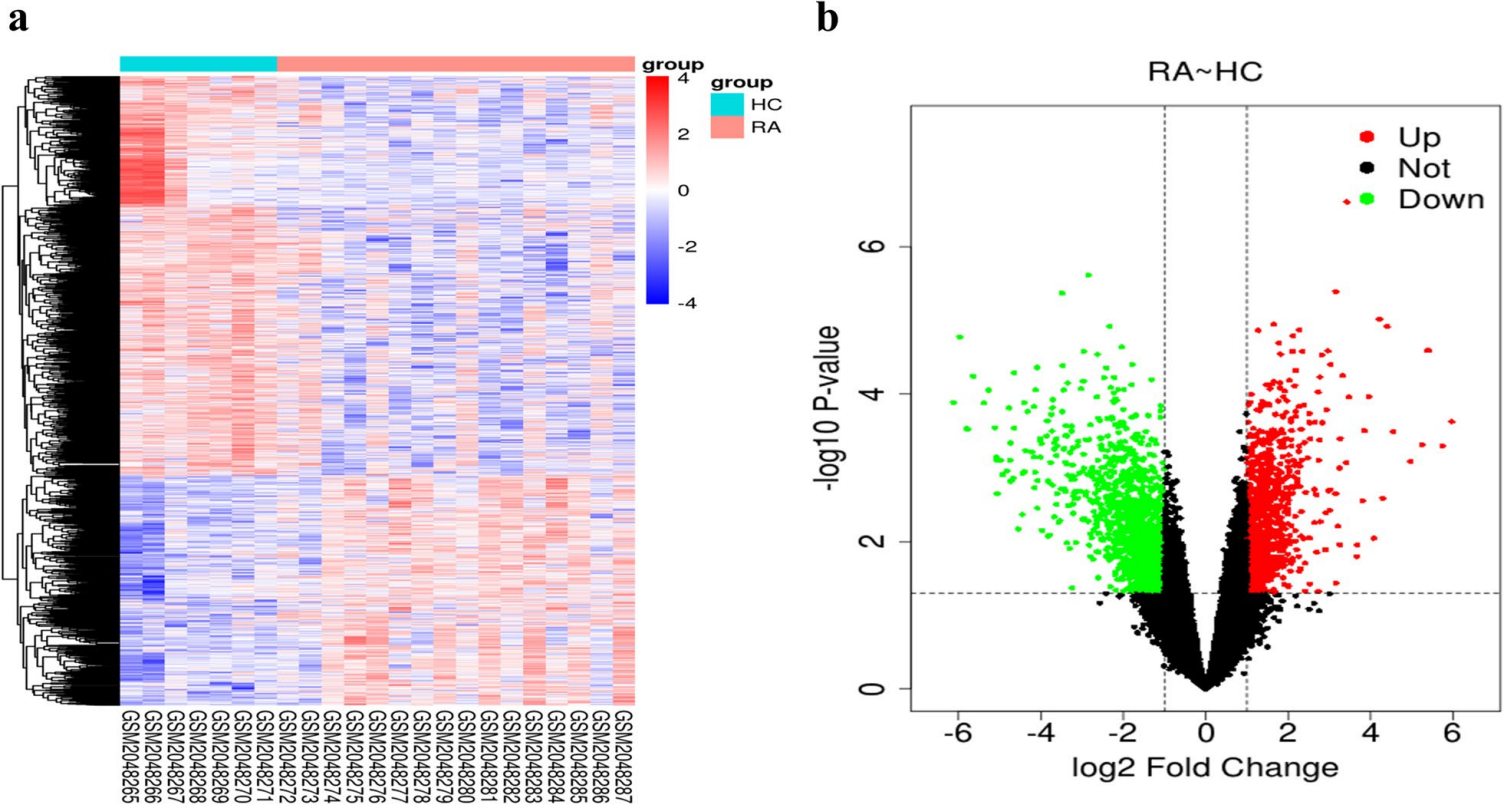
Differentially expressed genes (DEGs) in the synovial tissues of healthy controls and RA patients from GSE 77298. **a** Volcano plot of DEGs in the synovial tissues between control group and RA group. The red and green dots represent significantly upregulated and downregulated genes, respectively. **b** Heat maps of RA-related DEGs. The color from blue to red reflects a trend from low expression to high expression

GSE77298 was downloaded to analyze the DEGs between RA patient samples and control samples. A total of 2691 DEGs were identified in RA patients, of which 985 were upregulated and 1706 were downregulated (Fig. 2a, b).

### PPI network and hub targets

**Fig. 3:**
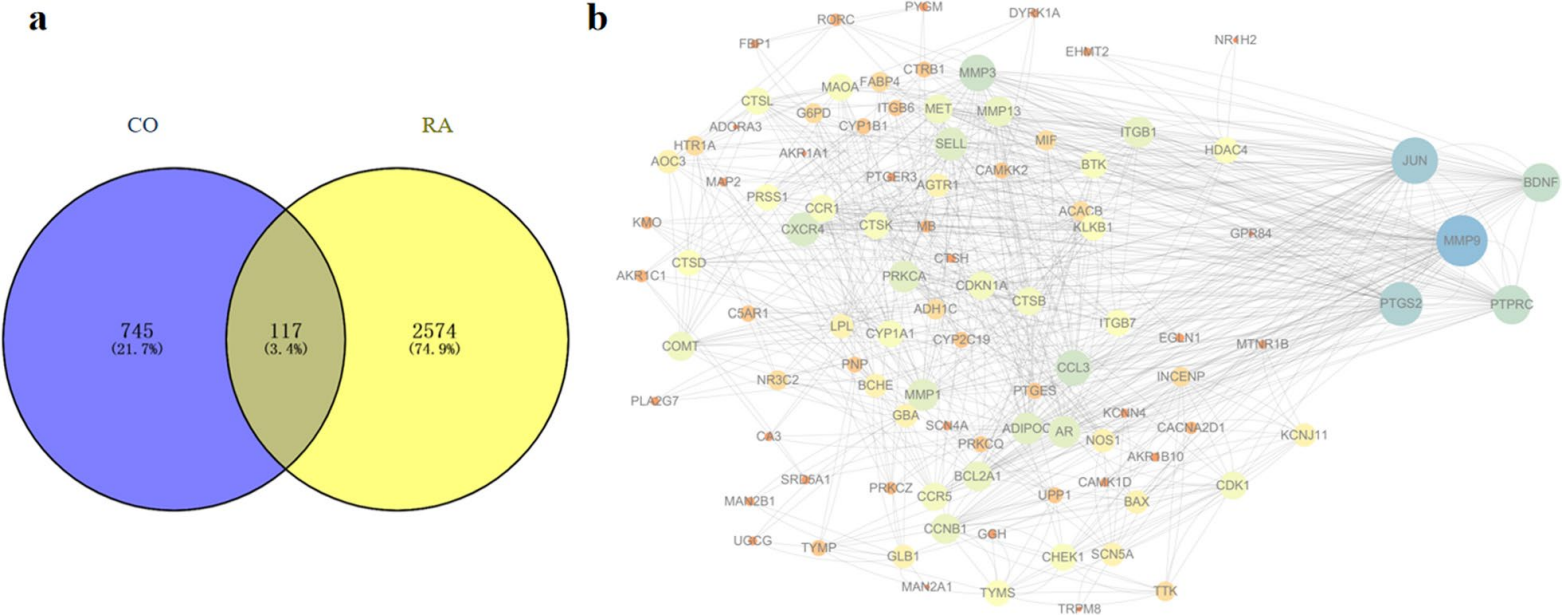
Network analysis of CO targets and RA-related DEGs. **a** Venn diagram of CO targets and RA-related DEGs. **b** The protein–protein interaction (PPI) network of common targets. The larger the circle, the more important the targets

According to the intersection from Venn diagram, we found 117 targets that may be related to the therapeutic effects of CO on RA (Fig. 3a). Next, we submitted the 117 overlapping genes into the STRING database to construct a PPI network. The results demonstrated that there were 117 nodes and 327 edges in the PPI network. The top 5 nodes ranked by the degree values were MMP9, JUN, PTGS2, BDNF, and PTPRC, which were the hub targets of CO for RA treatment (Fig. 3b).

### Ingredients-targets network analysis

**Fig. 4:**
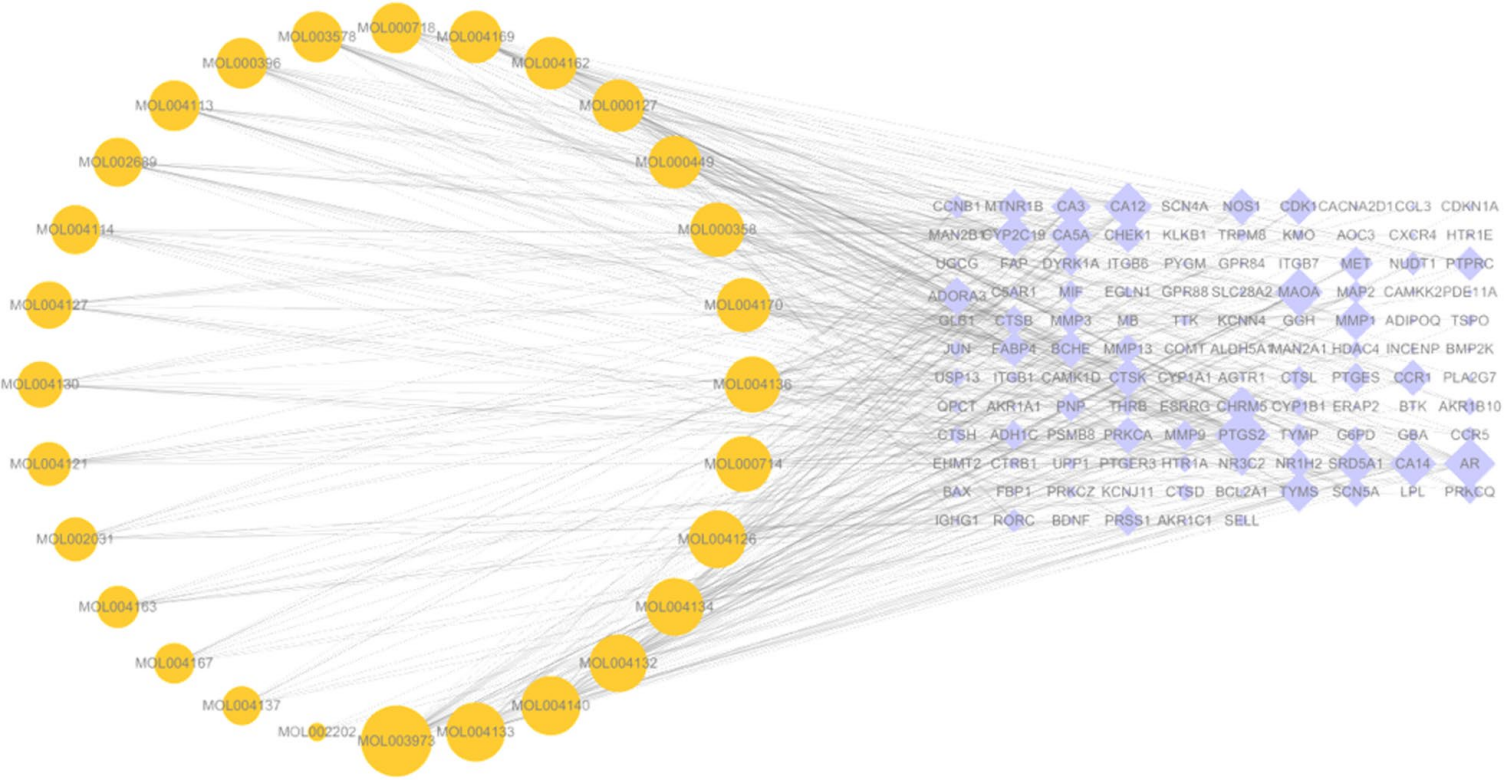
“Ingredients-Targets” network. The orange circles represent the active ingredients, and the purple prismatic represent the intersection targets

By using the Cytoscape software, we constructed the “ingredients-targets” network (Fig. 4). According to the network topology analysis, MOL003973 (caffeine), MOL004126 (curculigoside), MOL004133 (orcinol glucoside), MOL004134 (orcin), and MOL004140 (2,4-dichloro-5-methoxy-3-methylphenol) were the top five active ingredients with the highest degree values. These data suggested that CO might inhibit RA progression by these bioactive constituents to target multiple targets.

### GO and KEGG analysis

**Fig. 5:**
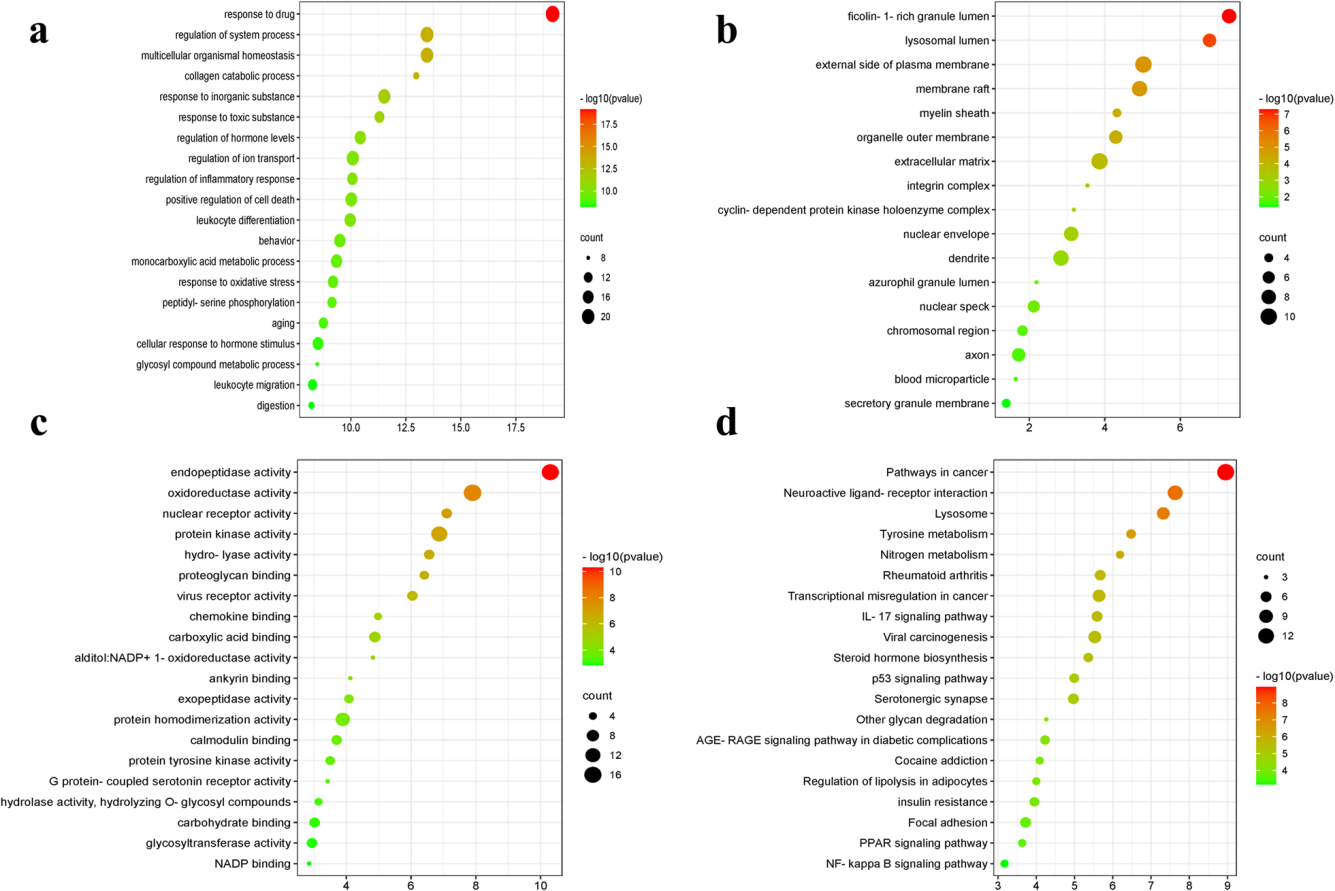
GO and KEGG pathway enrichment of common targets. **a** The GO terms including the top 20 biological processes (BP), cellular components (CC), and molecular functions (MF). **b** Bubble chart of the top 20 KEGG pathways. The dot size indicates the gene number, and the color indicates the p.adjust value

To explore the biological process and pharmacological mechanisms of CO against RA, these 117 overlapping targets were introduced into Metascape. According to *p* < 0.05, the top 20 terms were shown as bubble charts. The enrichment GO terms were regulation of inflammatory response, positive regulation of cytokine production, and membrane raft for biological process; ficolin-1-rich granule lumen and lysosomal lumen for molecular function; and endopeptidase activity and oxidoreductase activity for cellular component (Fig. 5a–c). KEGG enrichment analysis demonstrated that these targets were mainly involved in inflammation-related signaling pathways, including IL-17, P53, PPAR, and NF-κB (Fig. 5d). Therefore, CO might improve RA by regulating the inflammatory response.

### Molecular docking validation

**Fig. 6:**
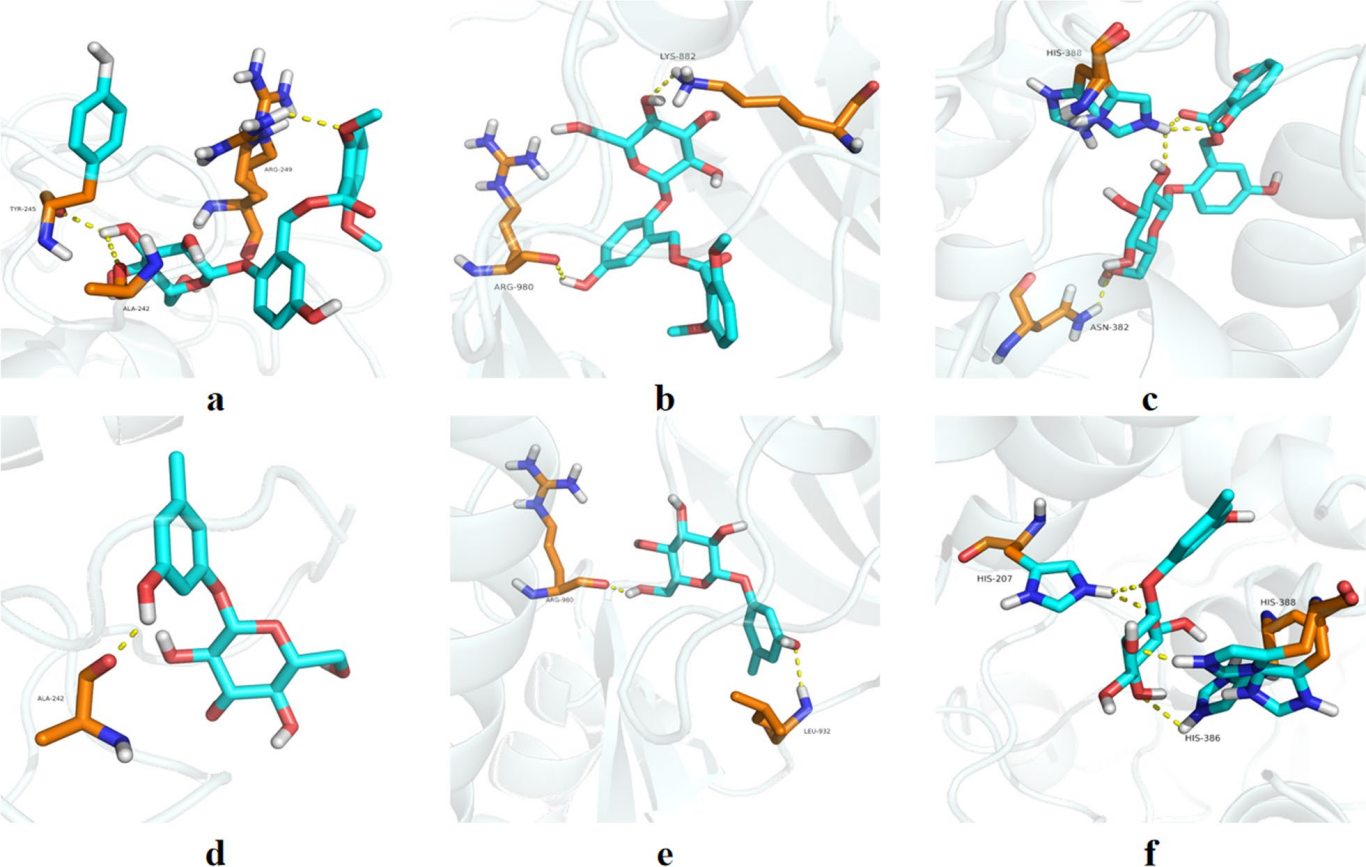
Representative images of molecular docking. **a**–**c** Curculigoside with MMP9, JUN, and PTGS2, respectively. **d**–**f** Orcinol glucoside with MMP9, JUN, and PTGS2, respectively

As described above, caffeine, curculigoside, orcinol glucoside, orcin, and 2,4-dichloro-5-methoxy-3-methylphenol were the main active ingredients for CO. These ingredients were then docked with the top five hub targets in PPI network. The 3D structures of hub genes including MMP9 (4h1q), JUN (5aep), PTGS2 (5f19), BDNF (1b8m) and PTPRC (5fmv) were obtained from the PDB database. The lower the binding energy, the stronger the binding activity. A binding energy < −7.0 kcal/mol represents a good binding affinity [24]. Curculigoside and orcinol glucoside had a strong binding activity to MMP9, JUN, and PTGS2, respectively. The detailed information is exhibited in Table 2. Curculigoside forms three hydrogen bonds with Tyr245, Ala242, and Arg249 in MMP9, two hydrogen bonds with Arg980 and Lys882 in JUN, and three hydrogen bonds with Asn382 and two hydrogen bonds with His388 in PTGS2. Orcinol glucoside forms one hydrogen bond with Ala242 in MMP9, two hydrogen bonds with Arg980 and Leu932 in JUN, and four hydrogen bonds with two hydrogen bonds with His207, His386, and His388 in PTGS2 (Fig. 6).

**Table 2:** Information on the molecular docking

Ingredients	Target protein	PDB ID	Docking score kcal/mol
Caffeine	MMP9	4H1Q	− 6.3
Caffeine	JUN	5AEP	− 6.3
Caffeine	PTGS2	5F19	− 6.4
Caffeine	BDNF	1B8M	− 4.4
Caffeine	PTPRC	5FMV	− 4.2
2,4-Dichloro-5-methoxy-3-methylphenol	MMP9	4H1Q	− 6.1
2,4-Dichloro-5-methoxy-3-methylphenol	JUN	5AEP	− 6.2
2,4-Dichloro-5-methoxy-3-methylphenol	PTGS2	5F19	− 6.2
2,4-Dichloro-5-methoxy-3-methylphenol	BDNF	1B8M	− 4.1
2,4-Dichloro-5-methoxy-3-methylphenol	PTPRC	5FMV	− 4.2
Orcinol glucoside	MMP9	4H1Q	− 8.0
Orcinol glucoside	JUN	5AEP	− 8.2
Orcinol glucoside	PTGS2	5F19	− 8.4
Orcinol glucoside	BDNF	1B8M	− 5.5
Orcinol glucoside	PTPRC	5FMV	− 5.3
Curculigoside	MMP9	4H1Q	− 8.3
Curculigoside	JUN	5AEP	− 9.3
Curculigoside	PTGS2	5F19	− 7.6
Curculigoside	BDNF	1B8M	− 6.2
Curculigoside	PTPRC	5FMV	− 6.5
Orcin	MMP9	4H1Q	− 6.7
Orcin	JUN	5AEP	− 5.5
Orcin	PTGS2	5F19	− 6.1
Orcin	BDNF	1B8M	− 4.7
Orcin	PTPRC	5FMV	− 4.3

### Experimental validation

#### Effect of CO on the viscera index in CIA rats

**Fig. 7:**
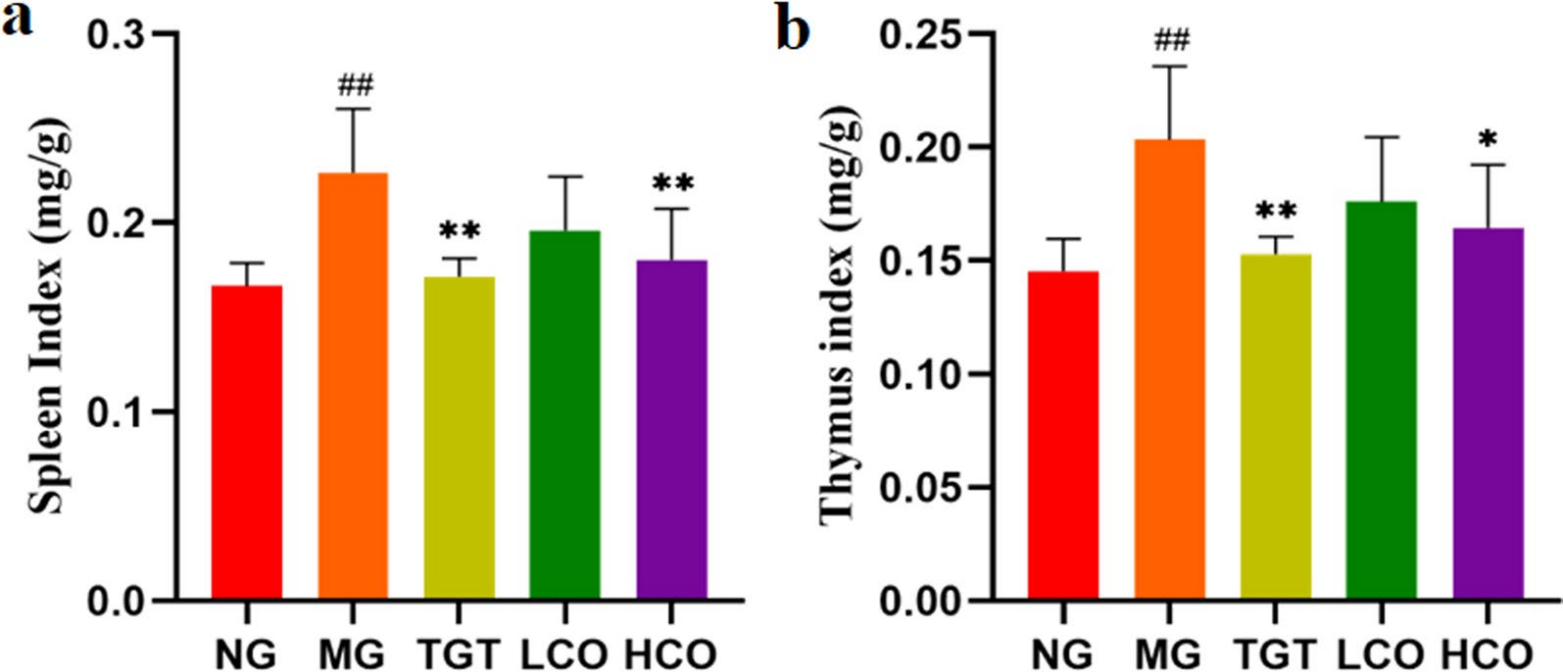
CO alleviated the viscera index in CIA rats (*n* = 8). **a** Spleen index. **b** Thymus index. ^##^*P* < 0.01 compared with NG; *P < 0.05, **P < 0.01 compared with MG

Spleen and thymus indices of rats were examined to evaluate the effects of CO on the immune organs (Fig. 7a, b). Compared with the normal group, rats in the model group displayed an obvious increase in spleen and thymus indices (*P* < 0.01). Compared with the model group, rats in the HCO and TGT groups showed markedly decreased spleen index (*P* < 0.01) and thymus index (*P* < 0.05).

### Effects of CO on the histopathological changes in CIA rats

**Fig. 8:**
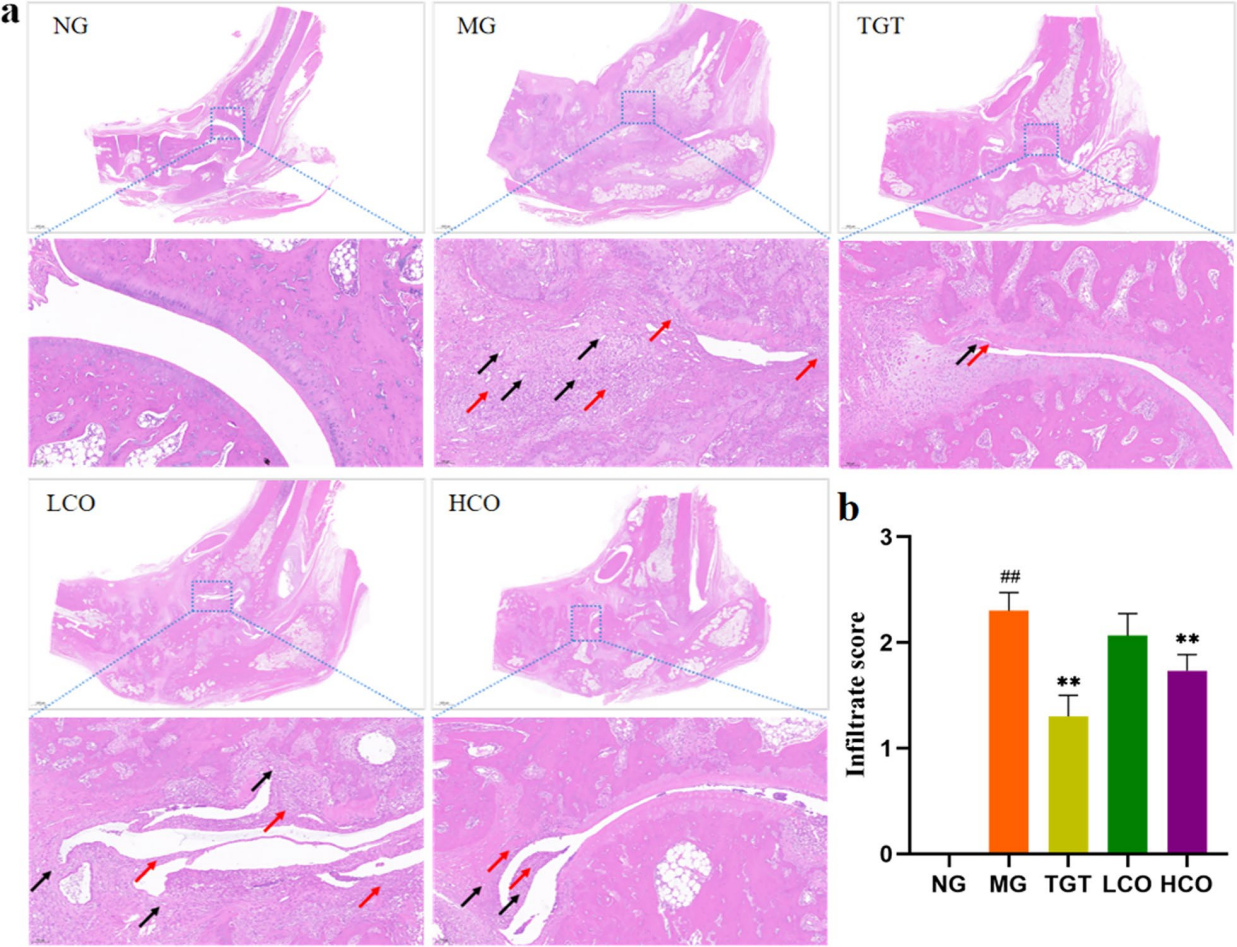
CO alleviated the RA symptoms in CIA rats. **a** Representative images of H&E staining for ankle joints (*n* = 3). **b** Histological score. Magnification, ×100. inflammatory cells: red arrows, pannus: black arrows. ^##^*P* < 0.01 compared with NG; **P < 0.01 compared with MG

As displayed by H&E staining, there was no pathological finding of arthritis for the rats in the normal group. In contrast, obvious inflammatory cell infiltration and pannus formation were found in CIA rats. However, the degree of inflammatory cell infiltration and pannus formation were significantly reduced in CIA rats treated with HCO or TGT (Fig. 8a). Semi-quantitative analysis showed a significant decrease in infiltration scores in HCO and TGT groups when compared with the model group (Fig. 8b).

**Fig. 9:**
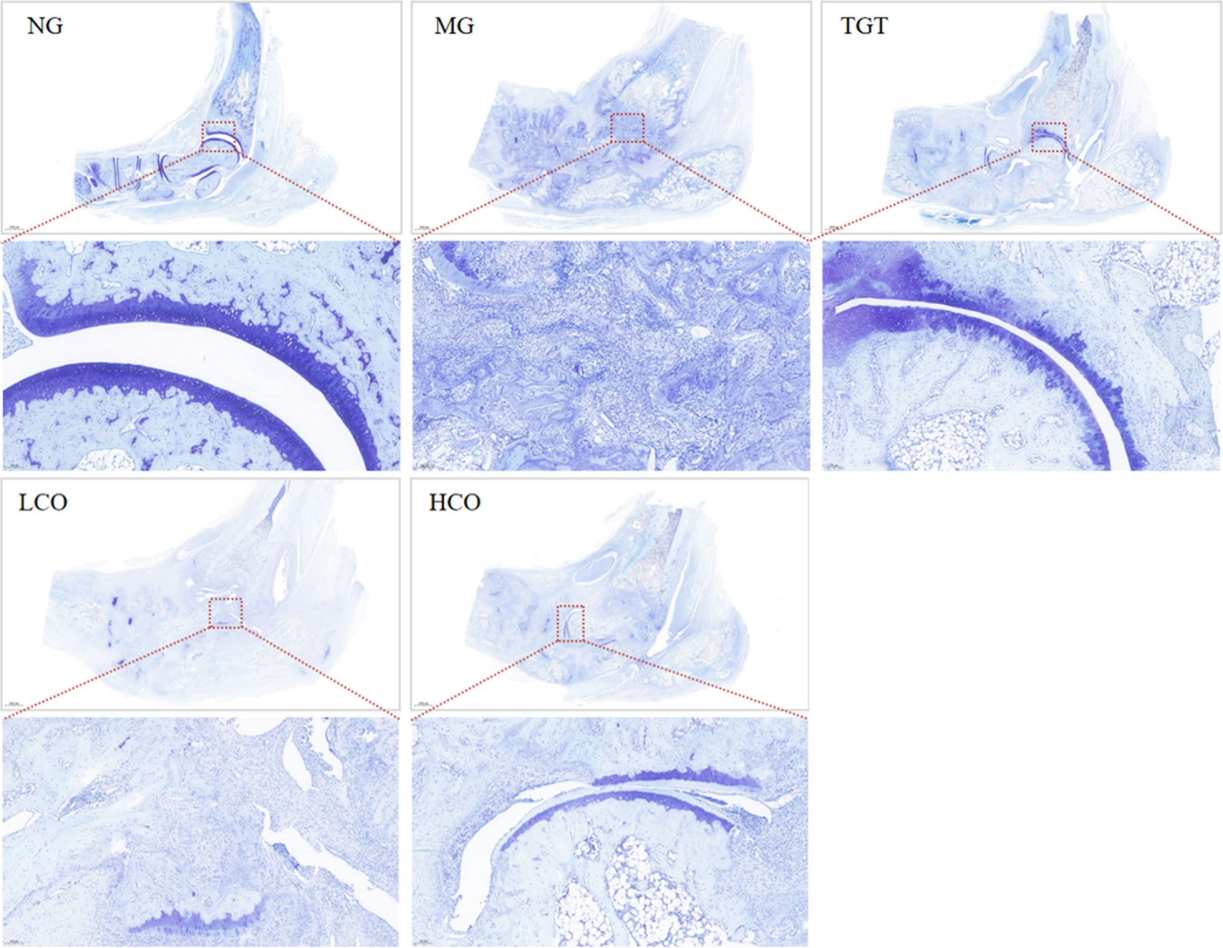
Representative images of toluidine blue staining for ankle joints (*n* = 3). Magnification, ×100

TB staining was performed to analyze the chondral injury of ankle joint. As shown in Fig. 9, the cartilage thickness was thinner, and inflammatory infiltration was fewer in the CIA rats than that in the normal rats. After treatment of TGT and CO, the cartilage damage was improved to varying degrees.

### Effects of CO on the expression of MMP9, JUN, and PTGS2 in CIA rats

**Fig. 10:**
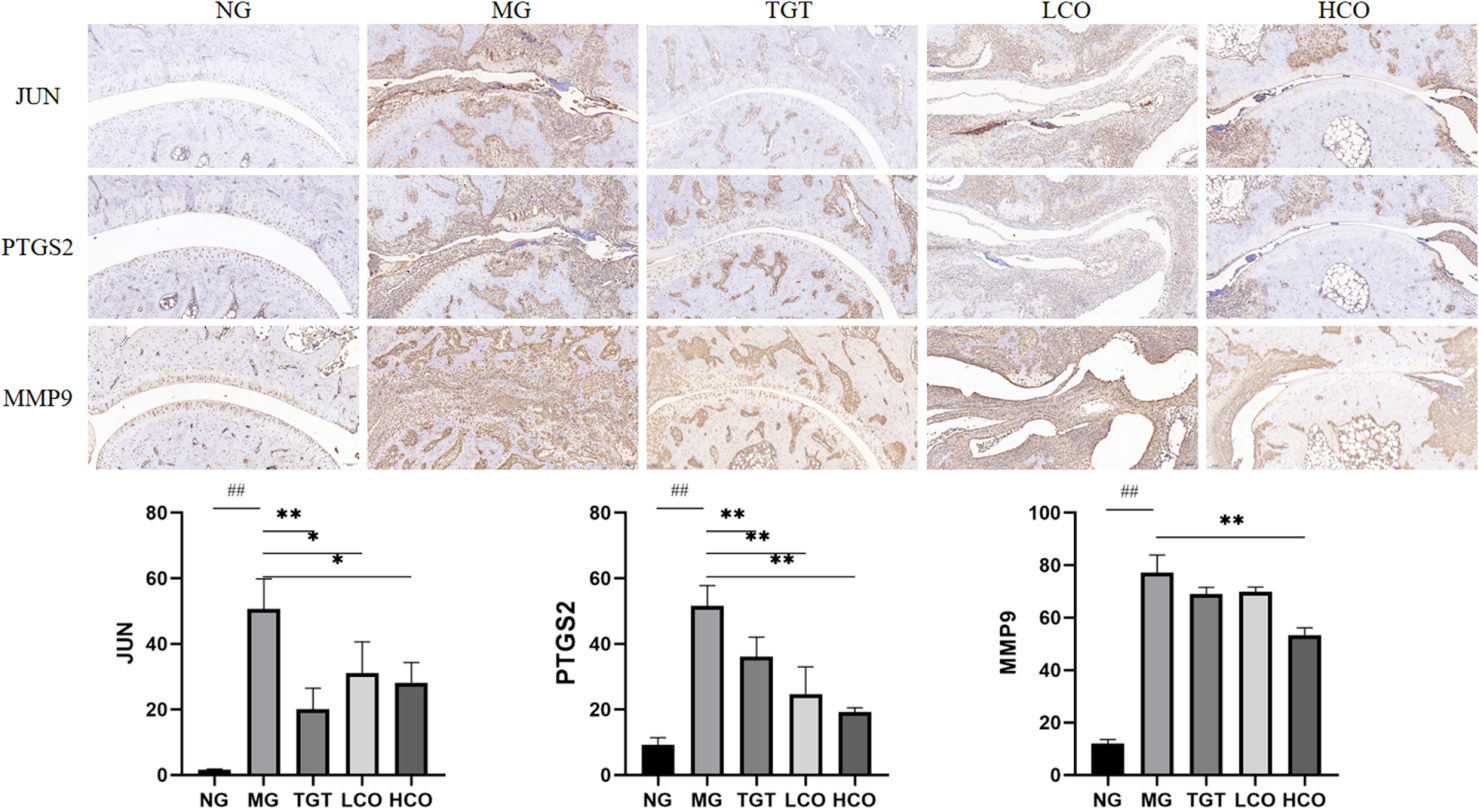
Immunohistochemical staining of JUN, PTGS2, and MMP9 expression in the ankle joint of CIA rats. Magnification, ×50. ^##^*P* < 0.01 compared with NG; *P < 0.05, **P < 0.01 compared with MG

Immunohistochemical analysis displayed that the expression of MMP9, JUN, and PTGS2 in the ankle joint tissues from the model group was drastically higher than that from the normal group (*P* < 0.01) (Fig. 10). However, the expression of MMP9, JUN, and PTGS2 was reduced after treatment with LCO, HCO, or TGT (*P* < 0.01).

## Discussion

In this study, network pharmacology combined with molecular docking and experimental validation was employed to investigate the active ingredients of CO and its possible pharmacological mechanisms against RA. Given the results of network analysis, 29 active ingredients and 5 hub targets participated in the anti-RA effect of CO.

The 29 active ingredients were mainly phenols and phenolic glycosides, triterpenes and triterpenoid glycosides, and alkaloids. Except caffeine, the other top four active ingredients, including curculigoside, orcinol glucoside, orcin, and 2,4-dichloro-5-methoxy-3-methylphenol are all phenolic and phenolic glycosides, which is consistent with the reported study that CO alcohol extracts are rich in phenols and phenolic glycosides [19]. Previous studies have indicated that curculigoside can alleviate RA symptoms by downregulating inflammation-related signaling pathways, such as NF-кB/NLRP3 [21] and JAK/STAT/NF-κB [22]. Orcinol glucoside has anti-osteoporosis [25], anti-perimenopausal depression [26], and anti-anxiety effects [27]. Therefore, curculigoside and orcinol glucoside might be the main bioactive compounds contributing to the anti-RA effect of CO.

In our present study, molecular docking results showed that curculigoside and orcinol glucoside have a strong binding ability to the hub targets (i.e., MMP9, JUN, and PTGS2) in the form of hydrogen bonds. PPI network analysis results indicated that MMP9, JUN, PTGS2, BDNF, and PTPRC were the major differential proteins. GO and KEGG analysis demonstrated that the potential targets of CO against RA were mainly related to inflammation responses [28, 29]. Thereafter, a CIA rat model was used to explore the therapeutic effects and mechanisms of CO in RA. Histopathological results showed that CO significantly inhibited inflammatory cell infiltration, pannus formation and cartilage damage. Thus, CO is a promising anti-RA candidate.

Matrix metalloproteinases (MMPs), a type of metalloproteinase, play an important role in the degradation of extracellular matrix related to tissue damage in RA [30]. An enhanced inflammatory response and increased expression of pro-inflammatory cytokines are significant pathological features in RA [31, 32]. Pro-inflammatory cytokines, especially tumor necrosis factor (TNF)-α and interleukin (IL)-1β, stimulate synovial fibroblasts to induce different matrix metalloproteinases (i.e., MMP1, MMP3, MMP9, and MMP13) and joint-destructive enzymes (i.e., cyclooxygenase-2 (COX-2)) in the joints [33, 34]. These enzymes can mediate cartilage degradation and bone erosion, leading to pain and joint destruction [35]. In this study, our results confirmed that CO inhibited the expression of MMP9 in CIA rats, suggesting that CO might suppress RA by targeting MMP9.

JUN, a member of activator protein 1 (AP-1), participates in the regulation of inflammatory process in RA by synergistic interaction with NF-κB to activate the pro-inflammatory cytokines [36, 37]. In addition, JUN can directly control the pro-inflammatory mediator COX-2 in macrophages, which can increase the production of prostaglandin E2 (PGE2) and ultimately result in the degeneration of cartilage and bone [38, 39]. Our immunohistochemical results indicated that JUN protein expression in the ankle joints was dose-dependently inhibited by CO, indicating that the inhibition of JUN is one of the mechanisms by which CO to treat RA.

PTGS2, also known as COX-2, is a key enzyme in prostaglandin biosynthesis and plays a crucial role in inflammatory responses [40]. PTGS2 is highly expressed in synovial mast cells of RA patients, which induces the activation of NF-κB signaling to increase inflammation in the body [41, 42]. In this study, it was found that CO downregulated the expression of PTGS2 in CIA rats, suggesting that the effect of CO on RA is associated with the inhibition of PTGS2.

In brief, our study indicated that CO alcohol extracts alleviated the infiltration of inflammatory cells and the formation of pannus in CIA rats. The action mechanism of CO against RA might be related to the downregulation of MMP9, JUN, and PTGS2 expression. Curculigoside and orcinol glucoside are the main bioactive compounds, which have a strong binding ability to hub targets (i.e., MMP9, JUN, and PTGS2) in the form of hydrogen bonds.

## Conclusion

In this work, active ingredient screening, potential target prediction and pathway analysis were combined to explore the potential active ingredients and targets of CO against RA. Curculigoside and orcinol glucoside were identified as the main bioactive compounds, and MMP9, JUN, and PTGS2 were confirmed as the potential targets for CO to treat RA. Interestingly, the content of curculigoside and orcinol glucoside in pCO is higher than that of CO according to our previous research, respectively. Further studies are warranted to investigate the effects of curculigoside and orcinol glucoside on RA.

## Data Availability

The datasets used and/or analyzed during the current study are available from the corresponding author on reasonable request.

## Abbreviations

RA: Rheumatoid arthritis
CO: Curculigo orchioides
TCSMP: Traditional Chinese Medicine Systems Pharmacology
PPI: Protein–protein interaction
GO: Gene ontology
KEGG: Kyoto Enrichment of Genes and Genomes
CIA: Collagen-induced arthritis
H&E: Hematoxylin and eosin
TB: Toluidine blue
MMP9: Matrix metallopeptidase 9
JUN: Transcription factor AP-1
PTGS2: Prostaglandin-endoperoxide synthase 2
BDNF: Brain-derived neurotrophic factor
PTPRC: Receptor-type tyrosine-protein phosphatase C
IL-1β: Interleukin 1β
TNF-α: Tumor necrosis factor α
COX-2: Cyclooxygenase-2
AP-1: Activator protein 1
DAB: 3,3-Diaminobenzidine
SD: Standard deviation
ANOVA: One-way analysis of variance
